# Factors influencing psychological distress during a disease epidemic: Data from Australia's first outbreak of equine influenza

**DOI:** 10.1186/1471-2458-8-347

**Published:** 2008-10-03

**Authors:** Melanie R Taylor, Kingsley E Agho, Garry J Stevens, Beverley Raphael

**Affiliations:** 1 Science of Mental Health and Adversity Unit, School of Medicine, University of Western Sydney, Australia

## Abstract

**Background:**

In 2007 Australia experienced its first outbreak of highly infectious equine influenza. Government disease control measures were put in place to control, contain, and eradicate the disease; these measures included movement restrictions and quarantining of properties. This study was conducted to assess the psycho-social impacts of this disease, and this paper reports the prevalence of, and factors influencing, psychological distress during this outbreak.

**Methods:**

Data were collected using an online survey, with a link directed to the affected population via a number of industry groups. Psychological distress, as determined by the Kessler 10 Psychological Distress Scale, was the main outcome measure.

**Results:**

In total, 2760 people participated in this study. Extremely high levels of non-specific psychological distress were reported by respondents in this study, with 34% reporting high psychological distress (K10 > 22), compared to levels of around 12% in the Australian general population. Analysis, using backward stepwise binary logistic regression analysis, revealed that those living in high risk infection (red) zones (OR = 2.00; 95% CI: 1.57–2.55; p < 0.001) and disease buffer (amber) zones (OR = 1.83; 95% CI: 1.36–2.46; p < 0.001) were at much greater risk of high psychological distress than those living in uninfected (white zones). Although prevalence of high psychological distress was greater in infected EI zones and States, elevated levels of psychological distress were experienced in horse-owners nationally. Statistical analysis indicated that certain groups were more vulnerable to high psychological distress; specifically younger people, and those with lower levels of formal educational qualifications. Respondents whose principal source of income was from horse-related industry were more than twice as likely to have high psychological distress than those whose primary source of income was not linked to horse-related industry (OR = 2.23; 95% CI: 1.82–2.73; p < 0.001).

**Conclusion:**

Although, methodologically, this study had good internal validity, it has limited generalisability because it was not possible to identify, bound, or sample the target population accurately. However, this study is the first to collect psychological distress data from an affected population during such a disease outbreak and has potential to inform those involved in assessing the potential psychological impacts of human infectious diseases, such as pandemic influenza.

## Background

Equine influenza (EI) is an acute, highly contagious viral disease which can cause rapidly spreading outbreaks of respiratory disease in horses and other equine species. It does not infect humans, but the virus can be physically carried on skin, hair, clothing, shoes, vehicles and equipment and through these means can be transferred to other horses. In addition, the windborne virus can be spread for distances up to eight kilometres [1].

### Australia's 2007 Outbreak

Australia's first outbreak of EI was confirmed on August 24^th ^2007. It spread quickly, but was successfully contained within areas of South East Queensland (Qld) and New South Wales (NSW). Although EI was not detected in other States and Territories, stringent disease control procedures were put in place across all States; which included an initial stand-still of all horse movements and subsequent controls, movement restrictions, and biosecurity requirements for many months. Colour-coded EI control zones were established within four weeks of the outbreak based on the level of disease/disease risk in Local Government Areas in NSW and Qld; these were adjusted as the disease spread, and each zone was subject to specific controls and restrictions. Controls were reviewed, revised and expanded as the disease spread, subsequent disease containment and control progressed, and policies were revised. These zones are summarized in Table 1. Further details of the outbreak, restrictions and zoning are available via the NSW Department of Primary Industries (NSW DPI) and Qld. Department of Primary Industries and Fisheries (DPI&F) websites [2,3]. Throughout the outbreak movement restrictions and biosecurity requirements remained in place, and no (or very limited) horse movement was ever allowed from higher risk zones to lower risk zones.

**Table 1:** Summary of EI control zoning, (based on NSW DPI zoning descriptions).

**Infection level/risk**	**Zone colour**	**Infection level**	**States/Regions affected**	**Summary of actions during outbreak**
High	Purple	Restricted area: High initial infection	NSW: around Sydney and stretching up North West to include Hunter Valley area	Initially subject to high degrees of control and quarantining. Region of relatively high/dense horse population. Later in outbreak disease rate was so high that disease in this area was left to run its course; quarantine was lifted and movement restrictions *within *the zone were relaxed.
	
	Red	Restricted area: High risk of disease	NSW: largely around purple zone, and South East Qld.	Very high levels of movement control and restriction were maintained throughout, infected areas were quarantined, and high levels of disease monitoring were maintained.
	
	Amber	Restricted area: Buffer zones	NSW, Qld.	High levels of movement control and monitoring were in place throughout outbreak. Control and containment using vaccination was initiated in these zones first.
	
	Green	Protected Area: Uninfected in NSW/Qld	NSW, Qld.	High levels of movement control and vigilance were in place throughout, although there was no infection detected.
	
Low	White	Uninfected	Vic., ACT, Tas., NT, SA, WA.	Initial movement standstill, general surveillance throughout outbreak and increasing easing of movement controls within and between States over time.

The disease outbreak peaked in late September/early October 2007, and then declined as successful containment and eradication strategies were progressed. The last new infections of EI were reported in NSW and Qld in December 2007. In total approximately 6,000 properties and 47,000 horses were infected in NSW and at least 3,000 properties were infected in Qld. Current data from disease surveillance and monitoring indicates that no active infection is present in Australia and the expectation is that Australia will be declared EI-free by the end of June 2008; if successful, Australia will be the only EI infected country in the world to have eradicated the disease.

The effects of EI and the disease containment strategy, like the horse industry itself, were varied and wide-ranging; impacting differentially on horse owners and those involved with the horse industry nationally. In terms of support to those affected, a range of government financial support and assistance was available to many of those affected within a short time of outbreak onset and financial and economic impact surveys were undertaken to provide feedback information to government [4,5]. The current study was conducted to gain additional complementary data to assess the impacts of EI on the social and emotional health and well-being of those affected. This paper reports data collected on non-specific psychological distress; however the full study covered many other aspects, such as adherence to biosecurity requirements, effects of social isolation due to quarantine and the consequences of restricted horse movement and related activities, and sources of support and coping during the EI outbreak.

### Human response to other infectious animal disease outbreaks

Although EI is endemic in Europe and North America, and has occurred as an epidemic in many other countries, e.g. Japan, South Africa, Hong Kong, there does not appear to be any published studies of the human response or impacts to EI or the containment strategies used to control this disease. The best reported and documented research with respect to the impacts of infectious animal disease on people is the outbreaks of foot and mouth disease (FMD) in Europe in 2001, specifically in the UK and The Netherlands.

Like EI FMD is highly contagious, however, FMD is considerably more serious as it spreads to cloven-hoofed animals including cattle, sheep, pigs, and goats. During these FMD outbreaks an estimated 4 million livestock were slaughtered on 9,000 farms in the UK (including many healthy animals as part of 'contiguous' or preventative culling on farms neighbouring infected farms) and 270,000 were culled in The Netherlands. The impacts on people were both economic, through financial/business/tourism-related losses, and psychological, through the exposure to loss of livestock, culling, and massive funeral pyres; the latter affecting not just farmers and their families, but also the wider population through media images on the television and in newspapers [6-8]. In the UK higher 'caseness' as indicated by the GHQ(G) was found in farmers from 'badly infected' areas, although higher psychological morbidity generally, was reported in farmers from both badly infected and unaffected areas [9].

In a study of Dutch dairy farmers [10] around half of those whose animals were culled suffered from severe post-traumatic distress, (identified as a clinical level of distress (> 25) using the 15-item Impact of Events Scale), with this reducing to one in five for those where severe restrictions were imposed (but where no culling took place). Higher levels of symptoms were reported for older respondents and those with lower levels of education. In this same study differences in stress, psychological marginalization, and depression were reported for different disease control areas, i.e. culled-area, buffer-area, FMD-free area [11].

Within Australia, the psycho-social impacts of Ovine Johne's disease have been reported [12,8] in which grief, depression, and anxiety were profound in affected farming families, and the perceptions of the management control process were the cause of much of the distress. Government policies on quarantining and de-stocking farms were suspended due to mounting reports of severe emotional and social distress in farmers, rural families, and government employees implementing those policies. Further discussion of stress in emergency responders managing agricultural emergencies is considered in an Australian context in a recent paper by Jenner [13].

The role of the animal-human bond on disaster preparedness and response is key feature in human response to animal disease, and has been review by Hall et al. [8]. These authors report several aspects of relevance to the current study, including the increasing role of horses as companion animals as opposed to livestock or economic investments, and hence an increasing emotional attachment to horses; the complex and dynamic emotional relationship between farmers and their livestock; the emotional and practical implications of the animal-human relationship in disaster management, e.g. compliance with disaster management behaviours; and the impacts on veterinarians as first responders in disasters. These authors conclude that recognizing the mental health aspects of the animal-human bond is an important factor in public health approaches to disaster and can be critical in promoting the resilience of individuals and communities. Therefore, it follows that in an animal-centred disease outbreak, such as EI affecting horses, the potential disruption of the animal-human bond, and the impact of policies restricting animal-human activities could have significant implications for the mental health and resilience of those affected.

### Psychological Distress

The main outcome measure in this study is non-specific psychological distress, as measured by the Kessler 10 (K10) [14]. The K10 was selected because it is a well-established and validated measure that is used widely in population research in Australia, it has been used in population health surveys in NSW [15], Victoria [16], South Australia [17], and Western Australia [18], as well as in National surveys conducted by the Australian Bureau of Statistics [19], and therefore State and National prevalence data are available as benchmarks for the current study. Scores from the K10 can be related to levels of intervention, with 'very high' psychological distress scores (> 30) equating to 'caseness' for a mental disorder, and high scores are strongly associated with current diagnosis of anxiety and depression using the Composite International Diagnostics Interview (CIDI) [20]. The K10 is also able to discriminate between DSM-IV cases and non-cases, and is felt to be an appropriate screening instrument for identifying likely cases of anxiety and depression in the population providing a strong marker for a possible mental health disorder [21-23].

In the most recent (2007) data from the NSW Adult Population Health Survey the combined proportion of the population reporting 'high' or above psychological distress (22–50) is 12.1% [24]. In addition, recent data collected in rural communities suggests that these figures may be slightly higher in rural-dwellers with 'very high' psychological distress of 5% reported in one study [25] and 13.4–13.8% for combined 'high'/'very high' psychological distress in another [26]. These findings are of relevance in the current study as it would be expected that horse-ownership would be linked to rural and peri-urban residency.

## Methods

### Survey design and sampling

The questionnaire was designed for online completion to expedite data gathering whilst the EI outbreak was occurring. Questionnaire content was reviewed by subject matter experts, including a small group of public health professionals in NSW Health, some of whom had been involved in aiding the NSW DPI in disease control management, a NSW DPI Local District Control Centre Controller who was responsible for leading control activities, and representatives of the Australian Horse Industry Council (AHIC). Ethics approval for the study was obtained through the University of Western Sydney ethics committee.

Horse owners, and those involved in the horse industry were invited to take part in the study via an e-mail alerting service administered by AHIC; using the national Horse Emergency Contact Database (HECD). The HECD had been established before the EI outbreak and was used as a network to contact and inform horse-owners during emergencies, such as bushfires, and disease outbreaks, and had been used previously by AHIC for collecting financial impacts information relating to EI earlier in the outbreak. This alerting service was used regularly during the EI crisis to update registrants with government support agency communications and general industry news and support information. Approximately 8,000 addressees were registered on the HECD; most were individuals, but also included were industry associations, pony clubs, and horse groups that would forward information to their own memberships nationally. Horse owners in NSW were encouraged by the NSW DPI to register on the HECD to receive up to date information.

The initial invitation to participate was sent to those registered on the HECD on 14 November 2007 (Week 12 of the outbreak). The survey remained open until 7 January 2008 (Week 21 of the outbreak) and date of completion was recorded with each respondent's data.

### Survey content and outcome measures

The full survey comprised 166 questions, covering a wide range of subject areas; those reported here include demographic information, i.e. gender, age category, number of children, highest level of educational qualification, and State/Territory of residence. In addition, respondents were asked about the nature of their current main involvement with horses (i.e. their industry sector), for example breeding, equestrian, recreational; whether their primary source of income was linked to a horse-related industry, and their current colour-coded EI control zone.

The main outcome measure reported in this paper is non-specific psychological distress as measured by the K10. This measure comprises 10 questions that ask respondents how often they have experienced certain symptoms during the preceding four weeks and responses are scored on a scale of 1 to 5 depending on how frequently each symptom is experienced, where 1 = 'none of the time', and 5 = 'all of the time'. Thus, a minimum score is 10, indicating no psychological distress, and a maximum score is 50, indicating the most severe level of psychological distress. Scores on the K10 are subsequently categorized into four levels: low (scores of 10–15); moderate (scores of 16–21); 'high' (scores of 22–29) and 'very high' (scores of 30–50) [27].

### Statistical methods

Statistical analyses were undertaken using STATA, version 9.2 (2004; Stata Corporation, College Station, TX, USA). Exploratory data analysis was conducted using frequency distributions for categorical variables. In the logistic model, a binary coding of psychological distress was used in which high psychological distress was a combination of 'high' + 'very high' levels of psychological distress = 1 (i.e. K10 scores of 22 or greater) and low psychological distress was a combination of 'low' + 'moderate' levels of psychological distress = 0 (i.e. K10 scores of 21 or less). Simple binary logistic regression and backward stepwise multiple logistic analyses were performed to identify factors influencing high psychological distress. All variables were entered into the model initially, with the least significant variables removed one at a time until only significant variables associated with values of p ≤ 0.05 remained. All statistical tests were two-tailed.

## Results

### Sample details

Details of the study sample are presented in Table 2. In total, 2,760 respondents completed the online survey, and of these 15% were male and 84% were female. More than a half of the sample (58.9%) had no children. A total of 40.2% of the respondents had a tertiary level educational qualification.

**Table 2:** Sample description (n = 2760)

		**n**	**%**
**Gender (n = 2736)**	Male	410	14.9
	Female	2326	84.3

**Age category (n = 2740)**	Under 16	36	1.3
	16–24	224	8.1
	25–34	482	17.5
	35–44	908	32.9
	45–54	771	27.9
	55–64	277	10.0
	65–74	39	1.4
	75+	3	0.1

**Number of children (n = 2700)**	0	1625	58.9
	1	436	15.8
	2	447	16.2
	3	136	4.9
	4+	56	2.0

**Educational qualifications (n = 2721)**	None	65	2.4
	School certificate	407	14.8
	HSC	394	14.3
	TAFE/Vocational	746	27.0
	University/Tertiary	1109	40.2

**Australian State/Territory (n = 2748)**	NSW	1303	47.2
	Qld.	556	20.1
	ACT	38	1.4
	NT	4	0.1
	Vic.	562	20.4
	Tas.	68	2.5
	SA	160	5.8
	WA	57	2.1

**Industry sector (n = 2758)**	Recreational	819	29.7
	Harness racing	139	5.0
	Thoroughbred racing	65	2.4
	Equestrian	734	26.6
	Stabling/Agistment	49	1.8
	Veterinary/health	47	1.7
	Breeding/Stud	429	15.5
	Farrier	22	0.8
	Commercial	195	7.1
	Multiple sectors	199	7.2
	Other	60	2.2

**EI control zone (n = 2668)**	White Zones	836	30.3
	Green Zones	335	12.1
	Amber Zones	320	11.6
	Red Zones	621	22.5
	Purple Zone	556	20.1

**Income linked to horse-related industry (n= 2740)**	Yes	632	22.9
	No	2108	76.4

Total count = 2760 unless otherwise given in brackets

Just under half the sample (47%) was from NSW and respondents from Qld. and Victoria (Vic) comprised a further 40% of the sample (20% from each State). Thirty percent of respondents were in uninfected white zones in States other than NSW and Qld, and 22% were from the restricted high EI risk red zones in NSW and Qld.

Around three quarters of the sample (73%) were from three industry sectors; recreational, equestrian, and breeding/stud sectors (30%, 27%, and 16%, respectively). The majority of respondents (76%) reported that their main source of income was not linked to a horse-related industry.

The prevalence of the four levels of psychological distress for the whole sample during the equine influenza outbreak; were 39% of respondents reporting 'low', 27% reporting 'moderate' 20% reporting 'high' and 14% reporting 'very high' levels of psychological distress. Table 3 shows the proportion of respondents reporting each level of psychological distress for the main socio-demographic survey variables.

**Table 3:** Prevalence of psychological distress by sample characteristics

		**Psychological Distress Level (K10 Score)**					
		**Low** **(10–15)**	**Moderate** **(16–21)**	**High** **(22–29)**	**Very high** **(30–50)**	**Total (%)**	**p-value**

**Gender**	Male	44.5	23.4	19.3	12.8	100	0.174
	Female	38.3	27.4	20.3	14.0	100	

**Age category**	Under 16	25.9	37.0	29.6	7.4	100	< 0.001
	16–24	29.9	27.2	21.7	21.2	100	
	25–34	35.3	29.9	21.1	13.7	100	
	35–44	36.8	26.7	21.5	15.1	100	
	45–54	42.7	24.6	20.4	12.4	100	
	55–64	49.6	27.1	14.8	8.6	100	
	65–74	60.0	20.0	2.9	17.1	100	
	75+	66.7	33.3	0.0	0.0	100	

**Number of children**	0	38.3	28.2	20.5	13.1	100	0.297
	1	37.5	24.7	20.0	17.8	100	
	2	42.4	23.5	20.4	13.8	100	
	3	42.0	27.7	16.0	14.3	100	
	4+	44.2	30.8	19.2	5.8	100	

**Educational qualifications**	None	32.7	23.6	25.5	18.2	100	0.161
	School certificate	34.7	26.4	21.7	17.2	100	
	HSC	39.7	24.6	22.1	13.6	100	
	TAFE/Vocational	40.4	27.4	17.1	15.2	100	
	University/Tertiary	40.1	27.7	20.6	11.6	100	

**Australian State/Territory**	NSW	36.0	27.8	21.6	14.5	100	< 0.001
	Qld.	32.0	26.2	22.5	19.4	100	
	ACT	46.0	35.1	18.9	0.0	100	
	NT	100.0	0.0	0.0	0.0	100	
	Vic	47.6	23.8	17.8	10.8	100	
	Tas.	47.5	30.5	13.6	8.5	100	
	SA	47.2	29.9	15.0	7.9	100	
	WA	66.0	21.3	8.5	4.3	100	

**Industry sector**	Recreational	44.9	27.5	17.4	10.2	100	0.004
	Harness racing	43.4	27.4	14.2	15.1	100	
	Thoroughbred racing	38.8	28.6	18.4	14.3	100	
	Equestrian	41.6	26.2	18.6	13.6	100	
	Stabling/Agistment	27.8	19.4	27.8	25.0	100	
	Veterinary/health	39.5	30.2	14.0	16.3	100	
	Breeding/Stud	30.5	28.3	25.1	16.0	100	
	Farrier	23.8	28.6	28.6	19.1	100	
	Commercial	33.7	26.0	23.1	17.2	100	
	Multiple sectors	34.1	25.4	26.0	14.5	100	
	Other	33.3	22.2	22.2	22.2	100	

**EI control zone**	White Zones	48.8	25.7	16.2	9.3	100	< 0.001
	Green Zones	42.3	23.4	19.2	15.1	100	
	Amber Zones	32.3	28.9	23.5	15.3	100	
	Red Zones	32.2	27.1	22.6	18.2	100	
	Purple Zone	35.0	29.4	21.7	13.9	100	

**Income horse-related**	Yes	28.2	23.5	27.6	20.7	100	< 0.001
	No	42.4	27.7	18.1	11.8	100	

The chi-square test was used to estimate p-values

The greatest prevalence of 'very high' psychological distress was reported for those respondents in the 16–24 age group (21.2%), and the lowest prevalence was reported by those in the 55–64 age group and those under 16 (8.6% and 7.4%, respectively). With regard to the remaining socio-demographic variables the highest prevalence of 'very high' psychological distress were recorded for those respondents who were female, those with one child, and those with no formal educational qualifications.

The prevalence of 'very high' psychological distress was greater for respondents from Qld. (19.4%) with prevalence figures being slightly lower for respondents from NSW (14.5%) and lower again for respondents from Vic. (10.8%). The highest prevalence of 'very high' psychological distress was found for respondents in the red zones (18.2%) and lowest for those in the white zones (9.3%). Those whose incomes were linked to horse-related industry had a higher prevalence of 'very high' psychological distress as compared to those whose main income was not linked to a horse-related industry (20.7% and 11.8%, respectively).

**Figure 1:**
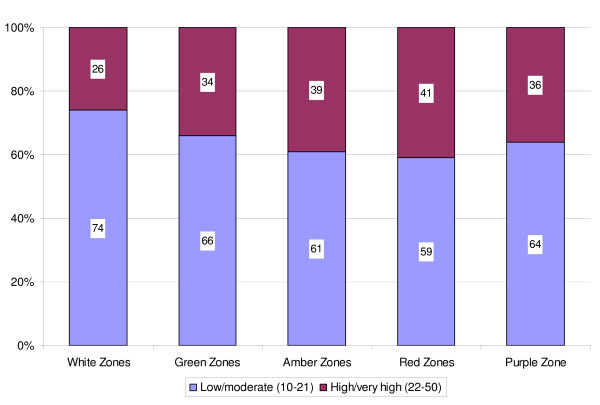
Psychological distress by EI control zone.

The four levels of psychological distress were combined in pairs ('low'/'moderate', and 'high'/'very high') to form a binary variable for subsequent statistical modelling. Figure 1 shows the prevalence of this binary high/low psychological distress variable by EI disease zones. Respondents in the red and amber zones reported higher prevalence of high psychological distress (41% and 39%, respectively) than those in the purple, green, and white zones (36%, 34%, and 26% respectively).

### Determinants of psychological distress

#### Univariate analysis

Table 4 shows the unadjusted and adjusted odds ratios (ORs) for the associations between high psychological distress (≥ 22) and socio-demographic variables.

**Table 4:** Factors associated with high psychological distress: Adjusted and Unadjusted Odds Ratios

		**Unadjusted OR**				**Adjusted OR**			
		
		OR	95% CI		p-value	OR	95% CI		p-value
**Gender**	Male	1.00	-	-	-	-	-	-	-
	Female	1.11	0.87	1.42	0.418	-	-	-	-

**Age category**	< 24	1.00	-	-	-	1.00			
	25–34	0.73	0.52	1.03	0.072	0.68	0.48	0.97	0.032
	35–44	0.79	0.58	1.07	0.131	0.74	0.54	1.02	0.066
	45–54	0.67	0.49	0.92	0.012	0.63	0.46	0.88	0.006
	55–64	0.42	0.28	0.63	< 0.001	0.34	0.23	0.52	< 0.001
	65+	0.31	0.13	0.73	0.008	0.28	0.11	0.67	0.005

**Number of children**	0	1.00	-	-	-	-	-	-	-
	1	1.20	0.95	1.52	0.127	-	-	-	-
	2	1.03	0.81	1.30	0.814	-	-	-	-
	3	0.86	0.57	1.29	0.464	-	-	-	-
	4+	0.66	0.35	1.25	0.202	-	-	-	-

**Education qualifications**	University/Tertiary	1.00	-	-	-	-	-	-	-
	TAFE/Vocational	1.00	0.81	1.24	0.983	-	-	-	-
	HSC	1.17	0.90	1.52	0.233	-	-	-	-
	School certificate	1.34	1.04	1.73	0.025	-	-	-	-
	None	1.63	0.94	2.83	0.081	-	-	-	-

**Australian State/Territory**	Other	1.00	-	-	-	-	-	-	-
	Vic.	1.57	1.10	2.25	0.013	-	-	-	-
	Qld.	2.83	2.00	4.00	< 0.001	-	-	-	-
	NSW	2.22	1.61	3.06	< 0.001	-	-	-	-

**EI control zone**	White Zones	1.00	-	-	-	1.00	-	-	-
	Green Zones	1.53	1.14	2.05	0.005	1.53	1.13	2.07	0.005
	Amber Zones	1.85	1.38	2.47	< 0.001	1.83	1.36	2.46	< 0.001
	Red Zones	2.01	1.58	2.55	< 0.001	2.00	1.57	2.55	< 0.001
	Purple Zone	1.61	1.26	2.07	< 0.001	1.53	1.19	1.97	0.001

**Income horse-related**	No	1.00	-	-	-	1.00	-	-	-
	Yes	2.19	1.80	2.67	< 0.001	2.23	1.82	2.73	< 0.001

Other = NT; ACT; Tas., SA and WA; Age < 24 = Under 16 and 16–24; OR = odds ratio; CI = confidence interval Multiple logistic regression analysis was used to adjust the ORs for the factors listed in the table.

Analysis by gender indicated a higher risk for high psychological distress for women as compared to men (unadjusted: OR = 1.11; 95% CI: 0.87–1.42), however this difference was not significant (p = 0.418). Analysis by age indicated a generally decreasing trend in risk of high psychological distress with age; respondents in the youngest age categories (under 24 years), had a higher risk of high psychological distress when compared to all other age groups. Relative to this youngest group respondents in the 55–64 year age category had the lowest risk of high psychological distress (unadjusted: OR = 0.42, 95% CI: 0.28–0.63; p < 0.001). Level of education was also associated with high psychological distress such that respondents with increasing levels of formal educational had lower risks of high psychological distress. Those with school certificate as the highest level of formal education were 1.34 times more likely to have high psychological distress compared to those with tertiary level qualifications (unadjusted: OR = 1.34, 95% CI: 1.04–1.73; p < 0.05). Those with no formal educational qualifications were 1.63 times more likely to have high psychological distress than those with university qualifications, however this result did not reach a level of statistical significance (p = 0.08).

Analysis by EI control zone indicated that respondents in red zones (unadjusted: OR = 2.01, 95% CI: 1.58–2.55; p < 0.001) and amber zones (unadjusted: OR = 1.85, 95% CI: 1.38–2.47; p < 0.001) were at greater risk of high psychological distress than those in purple zones (unadjusted: OR = 1.61, 95% CI: 1.26–2.07; p < 0.001) and green zones (unadjusted: OR = 1.53, 95%CI: 1.14–2.05; p = 0.005). In general, respondents in all NSW and Qld EI control zones were more than 1.53 times more likely to have high psychological distress than those in the white zones in other States. Analysis by State/Territory indicated that respondents who lived in Qld, NSW, and Vic. (unadjusted: OR = 2.83, 95% CI: 2.00–4.00; p < 0.001; unadjusted: OR = 2.22, 95% CI: 1.61–3.06; p < 0.001; and unadjusted: OR = 1.57, 95% CI: 1.10–2.25; p = 0.013, respectively) had a significantly higher risk of high psychological distress compared to those living in other States and Territories in Australia.

Respondents whose main source of income was from horse-related industry (unadjusted: OR = 2.19, 95% CI: 1.80–2.67; p < 0.001) were at a greater risk of high psychological distress than those whose main income was not linked to horse-related industry.

#### Multivariate analysis

As compared to < 24 age group, the following age groups: 24–34; 35–44; 45–54; 55–64 and 64+ were protective against high psychological distress (adjusted: OR = 0.63, 95% CI: 0.48–0.97; p = 0.032; adjusted: OR = 0.74, 95% CI: 0.54–1.02; p = 0.066; adjusted: OR = 0.63, 95% CI: 0.46–0.88; p = 0.006; adjusted: OR = 0.34, 95% CI: 0.23–0.52; p < 0.001; and adjusted: OR = 0.28, 95% CI: 0.11–0.67; p = 0.005, respectively).

Compared to respondents in white zones, respondents in red zones (adjusted: OR = 2.00, 95% CI: 1.5–2.55; p < 0.001) and amber zones (adjusted: OR = 1.83, 95% CI: 1.36–2.45; p < 0.001) were at greater risk of high psychological distress, as were respondents in purple zones (adjusted: OR = 1.53, 95% CI: 1.19–1.97; p < 0.001) and green zones (adjusted: OR = 1.53, 95% CI: 1.13–2.07; p = 0.005). When EI control zone was removed from the backward stepwise multiple logistics regression and Australian States/Territory was added, it was observed that, those in Qld, NSW and Vic. (adjusted: OR = 2.64, 95% CI: 1.85–3.76; p < 0.001; adjusted: OR = 2.13, 95% CI: 1.54–2.93; p < 0.001; adjusted: OR = 1.49, 95% CI: 1.04–2.15; p = 0.032, respectively) had a higher risk of high psychological distress than those in other States or Territories in Australia.

Respondents whose main source of income was from horse-related industry (adjusted: OR = 2.23, 95% CI: 1.82–2.73; p < 0.001) were at a greater risk of high psychological distress as compared to those whose income was not linked to horse-related industry.

## Discussion

The most salient finding was the extremely high prevalence of high psychological distress in horse owners and those involved in the horse industry during a serious horse disease epidemic; with just over one third (34%) reporting levels of psychological distress that might require some form of external intervention, and 40% of these (14% of the sample) reaching levels that may be considered indicative of 'caseness' for a DSM-IV disorder. The prevalence of 'very high' psychological distress in this sample was approaching five times the level reported in recent population health data for NSW [24]. Although this prevalence is very high, and there are some methodological reasons why this may be distorted (see study limitations section) it is certainly true that many of those impacted by EI, or the threat of EI, were subject to a wide range of acute stressors over a prolonged period, in a country where EI and such rigorous disease containment and control measures were previously unknown.

Analysis of psychological distress prevalence within the sample indicated that EI control zone was associated with psychological distress. Those in the areas where EI was present had higher risk of high psychological distress, furthermore, risks were higher in areas where EI was more active or threatening and the tightest levels of disease control were in place (i.e. red and amber/buffer zones). This finding suggests high levels of anticipatory anxiety. Interestingly, the risks of higher psychological distress in the purple zone (the region in NSW with the highest infection rate and earliest infections) were lower than in the red and amber areas. It is probable that during the timing of the study EI was more of a 'known' threat to those in the purple zone and there would have been some habituation to this risk; with many properties already infected or recovering, and restrictions eased due to the decision to let EI 'run its course' in this area at that time.

As disease control (and zoning) was controlled at a State level there is geographical overlap and co-linearity of the Australian State/Territory and EI control zone variables in the analysis; in the backward stepwise multiple logistic analyses excluding one made the other a significant factor. With regard to analysis by State, it is interesting to note the high levels of psychological distress reported in Victoria. Although Victoria remained EI-free throughout the crisis, those in Victoria were 1.57 times more likely to experience high psychological distress that those in the other uninfected States. There are probably a number of reasons for this effect: Victoria has a very extensive horse industry and is geographical closer to the infected States and disease-affected areas of NSW and Qld; there is also a high level of business interaction and physical movement of horses between Victoria and NSW and Qld; hence the level of proximal threat and the degree of disruption caused by disease control measures was probably experienced more widely in Victoria and may explain some of this effect. It should also be noted that although the remaining States were similarly uninfected, the overall prevalence of high psychological distress in horse owners from these States was still far higher than in the general population; those uninfected were not unaffected.

One of the other primary factors associated with high psychological distress was age. Those in the 16–24 year age category reported the highest levels of high psychological distress and analysis indicated that although prevalence and comparative risks of high psychological distress reduced from age 24 onwards, these reduced risks only became reliably statistically significant from age 45 onwards, and high psychological distress was certainly still a risk to those in the 35–44 year age category. This is interesting because in the general population psychological distress is generally found to peak around middle age (40s–50s). The study findings would suggest that younger people were particularly vulnerable and were coping less well with the consequences of EI. The reasons for this finding are not known, however, research literature suggests that younger people form stronger emotional attachments to animals [28], and they are also less likely to be resilient or practised, generally, when it comes to coping with adversity. From the general perspective of mental and physical health of younger people, it is interesting to consider the longer term consequences and potential burden of disease if these effects are enduring.

It is also interesting to note here the association of psychological distress with having children. Data in this study indicated that those with one child had a 1.2 times higher risk of high psychological distress than those with no children; and having three or more children appeared somewhat protective against high psychological distress. National statistics would support the suggestion that those with one child are generally younger adults and/or are 'young families' with a single younger child. In this study, 17.8% of respondents with one child reported 'very high' levels of psychological distress (K10 score = 30–50). Given these family circumstances such a finding may be a cause for concern.

The final main factor associated with high psychological distress was having an income linked to horse-related industry. Unsurprisingly, those with financial dependence on an industry facing such a crisis are likely to be significantly predisposed to high psychological distress. Nothing has been mentioned in this paper on the industry sector from which respondents had their main involvement with horses. These data were reported as part of the sample description to illustrate the wide range of industries affected by EI and the complexity of the affected population, and to provide information to aid interpretation of the findings. The nature of the potential psychological impacts of EI on those in different sectors is extremely diverse; from purely economic impacts, to loss of leisure pursuits and disruption of social networks, to loss of futures and missed opportunities in time, and many other possible impacts. Time, money and support will help most recover but it is possible that some people's mental and physical health will be permanently affected by EI and some will take many years to recover professionally if they choose to stay in these professions.

Given the level of psychological distress noted in the current study, it is interesting to consider the distress that might result from other epizootics, such as foot and mouth disease or avian influenza, and how this, in turn, might compare to the levels of distress resulting from human epidemics, such as SARS and H5N1/pandemic influenza. As mentioned earlier, foot and mouth disease in Europe resulted in high distress and PTSD in farmers. In relation to avian influenza, most research has focussed on risk perception and compliance with protective behaviours. A large European Union project on risk perception to avian influenza in Europe and Asia found moderate levels of risk perception generally, with higher levels of risk perception noted in Europe, and in females in most countries [29]. Considering distress and risk perception in relation to human epidemics; it is likely that psychological distress would be far greater, since these present a threat to human health and possibly death. Certainly data collected during and after SARS in Hong Kong found high levels of fear and PTSD in health care workers and hospital workers [30], and high levels of emotional disturbance in the general population [31]. Research in Canada found enduring psychological distress, up to two years following SARS, among health care workers in a hospital that treated SARS patients [32].

## Study limitations

This study had a number of limitations that should be considered when interpreting the data. Firstly, the target population; those affected by EI, is a complex, disparate, and unknown population and therefore it is difficult to comment accurately on the representativeness of the sample. All horse owners in Australia are not registered on a centralized database, or otherwise controlled, and as a result, it is not possible to know how extensive the database used to access horse owners (the HECD) was. However, at a national cross-industry sector level it is believed that this was the most extensive and efficient online route to access the target population, and the use of the database as a central communication facility during the EI crisis meant that this was likely to have been a focus for those affected during the epidemic.

Due to demographic bias in the sample, in particular, a greater proportion of women, and those with higher levels of education it is possible that there will be response bias in the data. The high proportion of women in the sample may be due to greater interest and participation in studies of this nature, but may also be indicative of higher levels of females in the target population, in particular in the main industry sectors represented in the data, i.e. recreational and equestrian. There are no official statistics on gender breakdown across horse industry sub-populations in Australia, but data indicate that the equestrian sector in the United States may comprise 80% women [33], so the gender bias may reflect a gender bias in the main industry sub-populations in our data. Research data often report higher levels of psychological distress in women in the general population, and therefore, the gender demographic bias in our study might have led to an elevation in the levels of psychological distress reported in this study. However, the absence of a significant gender effect in this study, and the close matching of relative levels of psychological distress in men and women with data from the Australian general population, suggests that EI, as an adversity, was exerting similar impacts on males and females. It is not possible to explain why there was an absence of a gender difference in the data. One possible explanation is that the timing of the study; around the height of the EI epidemic, and the high levels of psychological distress generally, reflected peak, acute levels in which gender differences were minimised and insignificant.

As with gender bias, it is hard to define the impacts of education level in the data. Unlike the (female) gender bias in the data, higher levels of education offer a protective effect (as identified in the univariate analysis). Therefore, this source of bias may have led to an under-reporting of high psychological distress. Again, it is not possible to define or quantify the extent of this.

Finally, the use of an online survey imposes potential limitations. It is probable that the study findings under-represent the responses of those in certain demographics, e.g. those who are less educated (as noted), those less affluent, and older respondents. Not all horse owners would have access to the internet, and online survey methodology is relatively uncontrolled, e.g. the sample was self-selected and therefore may be more prone to response bias than a sample that was randomly selected or otherwise controlled. Also, those experiencing higher distress may have been more motivated to respond. The extent of this response bias on the data cannot be accurately estimated, however, in anticipation of potential response bias, actions were taken to ensure that the study was presented to potential respondents in a way that would minimize such effects; e.g. the study was presented as independent of any industry group or government organization and it was clearly identified as a university research study. It was hoped that such presentation of the study would reduce political or self-interest motivation for completing the study.

## Conclusion

Despite some methodological limitations, this study was able to determine the psychological impact of Australia's first outbreak of equine influenza on a substantial sample of horse owners and those involved in horse-related industry. Study findings indicated that this affected population had highly elevated levels of psychological distress and that, although prevalence of high psychological distress was greater in infected EI control zones and States, elevated levels of psychological distress were experienced in horse-owners nationally, and not just in areas where equine influenza was present. Statistical analysis indicated that certain groups were more vulnerable to high psychological distress; specifically younger people, those with no formal educational qualifications, and those whose main income was linked to a horse-related industry.

Findings from this study generate further questions: What were the determinants of elevated psychological distress? Was it the risk of the disease itself, e.g. fear of the disease, or concern for horses? Was it the social and emotional impacts of disease control measures and restrictions, e.g. social isolation, quarantine, loss of freedom or control, stigma of being 'infected'? Was it loss of income or sporting aspirations? More importantly, how enduring is this elevated psychological distress, and what are the longer term mental or physical health consequences for those affected? The latter is of critical importance given the increased prevalence of high psychological distress reported in young people in this study. Some of these questions can be addressed using additional data collected in the wider study; however, the issue of enduring psychological distress will require further assessment.

## Competing interests

The authors declare that they have no competing interests.

## Authors' contributions

MT was involved in all aspects of the research project; design, conducting the research, data handling, exploratory analysis, drafting and editing the paper. GS, and BR assisted with the design of the study and provided input in all aspects of the review, data interpretation and editing of the paper. KA conducted the statistical analysis and contributed to the drafting and review of the paper.

## Pre-publication history

The pre-publication history for this paper can be accessed here:


